# Symmetry-Directed Engineering of Quantum Defects: Toward Monodispersed Organic Color Centers in Carbon Nanotubes

**DOI:** 10.1021/acsnano.6c07965

**Published:** 2026-10-15

**Authors:** Mengyue Wang, Jacob Fortner, Benjamin Barnes, Daichi Kozawa, Chee Fai Fong, Jeffrey A. Fagan, Yuichiro K. Kato, YuHuang Wang

**Affiliations:** † Quantum Optoelectronics Research Team, 426913 RIKEN Center for Advanced Photonics, Wako, Saitama 351-0198, Japan; ‡ Nanoscale Quantum Photonics Laboratory, RIKEN Pioneering Research Institute, Wako, Saitama 351-0198, Japan; § Chemical Physics Program, Institute for Physical Science and Technology, 1068 University of Maryland, College Park, Maryland 20742, United States; ∥ Department of Chemistry and Biochemistry, University of Maryland, College Park, Maryland 20742, United States; ⊥ Materials Science and Engineering Division, National Institute of Standards and Technology, Gaithersburg, Maryland 20899, United States; # Research Center for Materials Nanoarchitectonics, National Institute for Materials Science, Tsukuba, Ibaraki 305-0044, Japan; ∇ Maryland NanoCenter, University of Maryland, College Park, Maryland 20742, United States

**Keywords:** Carbon Nanotubes, Organic Color Centers, Quantum Defects, Spectroscopy, Cycloaddition, Single Photon Sources, Density Functional Theory

## Abstract

Achieving spectrally uniform single-photon emission is crucial for scalable quantum photonics, yet conventional approaches often yield broad and unpredictable emission. Here, we report on the selective creation of organic color centers (OCCs) on high-symmetry zigzag (10,0) single-walled carbon nanotubes via a [2 + 2] cycloaddition reaction with 4-cyclopentene-1,3-dione. Density functional theory predicts two possible OCC binding configurations separated by >200 meV, favoring the selective formation of a single OCC geometry. Through chirality sorting, hyperspectral photoluminescence imaging, and multi-step spectral screening, we isolate functionalized (10,0) nanotubes and observe a substantially narrower distribution of OCC emission wavelengths than similarly functionalized chiral nanotubes. Time-resolved photoluminescence and photon correlation measurements further confirm single-photon emission at room temperature. These results demonstrate divalent OCCs on symmetric nanotubes as promising quantum emitters, offering a route toward scalable atomically defined light sources for integrated photonics.

## Graphical abstract

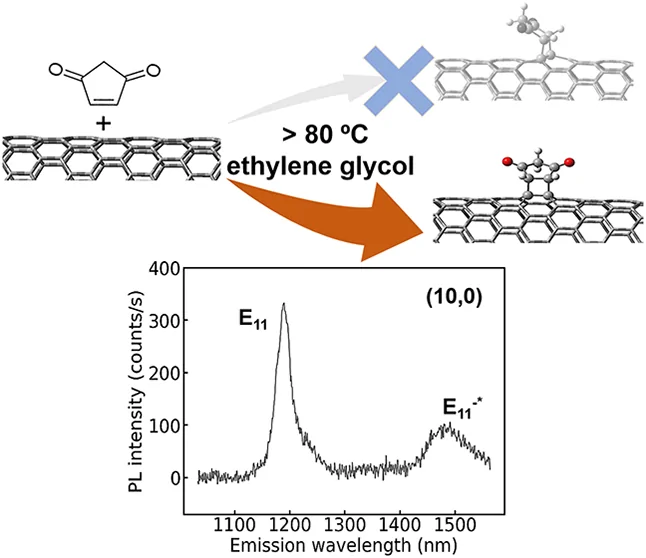

## Introduction

Organic color centers (OCCs) in single-walled carbon nanotubes (SWCNTs) have emerged as chemically tunable quantum emitters.^1–7^ Created through covalent functionalization, OCCs introduce sp^3^-defect sites into the sp^2^ carbon lattice, forming localized exciton traps^7^ that enable room-temperature single-photon emission.^3,8^ Compared with semiconductor quantum dots, which suffer from size and shape heterogeneity,^9^ or solid-state defect centers such as nitrogen-vacancy (NV) centers in diamond^10^ and defects in hexagonal boron nitride (hBN),^11,12^ OCCs offer a path to achieve atomically defined quantum emitters enabled by molecular design and covalent chemistry. Their emission in the shortwave infrared and telecom bands has further applications in bioimaging and quantum photonics.^13–15^

Molecular control provides versatile routes to tailor OCC emission properties. For example, modifications of the precursor molecules enable systematic wavelength tuning across the telecom band,^3,5,7,16^ add chemical selectivity,^17–19^ and provide reactive handles for further derivatization.^20,21^ Beyond precursor design, the functionalization environment has expanded from solution-phase to vapor-phase chemistry.^22^ By real-time monitoring and terminating the reaction at the single-defect level, single OCCs can be created on demand with controlled positions on air-suspended SWCNTs,^23^ and potentially further reconfigured by laser annealing.^24^ Together, these advances highlight the chemical versatility of OCCs for tuning emission properties and integrating new functionalities.

However, a central challenge remains in reducing the spectral inhomogeneity.^24,25^ Covalent reactions on the sp^2^ lattice typically produce multiple binding configurations, each with distinct optical transitions, resulting in broadened emission distributions.^26,27^ When sp^3^ defects are created on the surface of the SWCNT, they generally form in pairs to avoid radical generation. Density functional theory (DFT) predicts that paired sp^3^ defects can adopt either ortho or para configurations, each with three orientations relative to the nanotube axis, yielding six distinct structures.^27^ Time-dependent DFT (TD-DFT) further predicts that each structure has a different emission energy,^27^ directly linking configurational diversity to spectral broadening. This variability complicates efforts to achieve spectral uniformity and reproducibility.

Recent advances in [2 + 2] cycloaddition chemistry provide a strategy to address this challenge. In a [2 + 2] cycloaddition reaction, an enophile adds across a conjugated double bond on the SWCNT sidewall to form a four-membered ring, creating a well-defined covalent linkage. Unlike diazonium-based methods,^7,27^ the [2 + 2] reaction selectively produces ortho defect pairs, reducing the possible binding configurations from six to three.^28,29^ Here, we target high-symmetry zigzag (10,0) SWCNTs as a model system to demonstrate the creation of monodispersed OCCs through symmetry-directed defect engineering. In zigzag (*n*,0) nanotubes, the intrinsic mirror symmetry of the carbon lattice introduces degeneracy on defect orientations, thereby reducing configurational variants relative to lower-symmetry chiral nanotubes.^26^ Among zigzag chiralities, (10,0) provides a representative model system that preserves this high structural symmetry while maintaining a moderate diameter (∼0.8 nm), which enables convenient optical interrogation of its excitonic transitions in the near-infrared region. The mirror symmetry of (10,0) tubes render two of the three ortho (or para) configurations equivalent, leaving only two unique OCC structures. DFT calculations predict that these two configurations are separated by >200 meV in stability, suggesting that under thermal control, only the low-energy ortho­(+) configuration should dominate. Through this symmetry-directed defect engineering combined with chirality sorting and single-tube spectroscopy, we demonstrate that [2 + 2] cycloaddition of 4-cyclopentene-1,3-dione (CPD) produces divalent OCCs with reduced spectral diversity in (10,0) SWCNTs. Hyperspectral imaging and spectral screening steps isolate emission from CPD-(10,0) tubes, revealing a significantly narrower OCC emission wavelength distribution than that of functionalized (6,5) SWCNTs. Time-resolved photoluminescence (PL) and photon correlation measurements further confirm single-photon emission at room temperature. These results demonstrate divalent OCCs on symmetric nanotubes as a promising route to atomically define and reproduce quantum light sources for scalable photonic applications.

We first examine the possible OCC configurations formed by the [2 + 2] cycloaddition of CPD on (10,0) SWCNTs using DFT. Among the three precursors previously demonstrated for [2 + 2] cycloaddition,^28^ we focus here on CPD as the most chemically robust choice for generating monodispersed OCCs. Unlike *N*-methylmaleimide and maleic anhydride, which contain heteroatoms and can undergo side reactions such as ring opening or hydrolysis,^30,31^ CPD features a fused five-membered ring that enforces structural rigidity. This minimizes the likelihood of producing mixed defect chemistries (e.g., combinations of open- and closed-ring adducts) and ensures a single, well-defined divalent OCC motif. By strategically selecting CPD and applying it to a high-symmetry zigzag lattice, we establish a route that minimizes both chemical and structural sources of spectral diversity.

## Results and Discussion

Calculated reaction energies confirm the configurational selectivity achieved by this strategy, showing that both possible binding configurations are endothermic relative to the pristine nanotube but with strikingly different stabilities (Figure 1). The ortho­(+) configuration, in which the CPD aligns with the nanotube axis, is only 14.9 meV above the pristine state, whereas the tilted ortho­(++) configuration is 253.9 meV (≈10 *k*_b_T) higher in energy. This large energy separation (>200 meV) suggests that under thermal activation, the ortho­(+) product should dominate. Furthermore, time-dependent DFT calculations used to find the excited state energies show that the OCC transition associated with the ortho­(+) configuration is lower in energy than that of the ortho (++) configuration, with a redshift from the nanotube host’s bandgap transition *E*_11_ by 354 and 245 meV, respectively.

**Figure 1:**
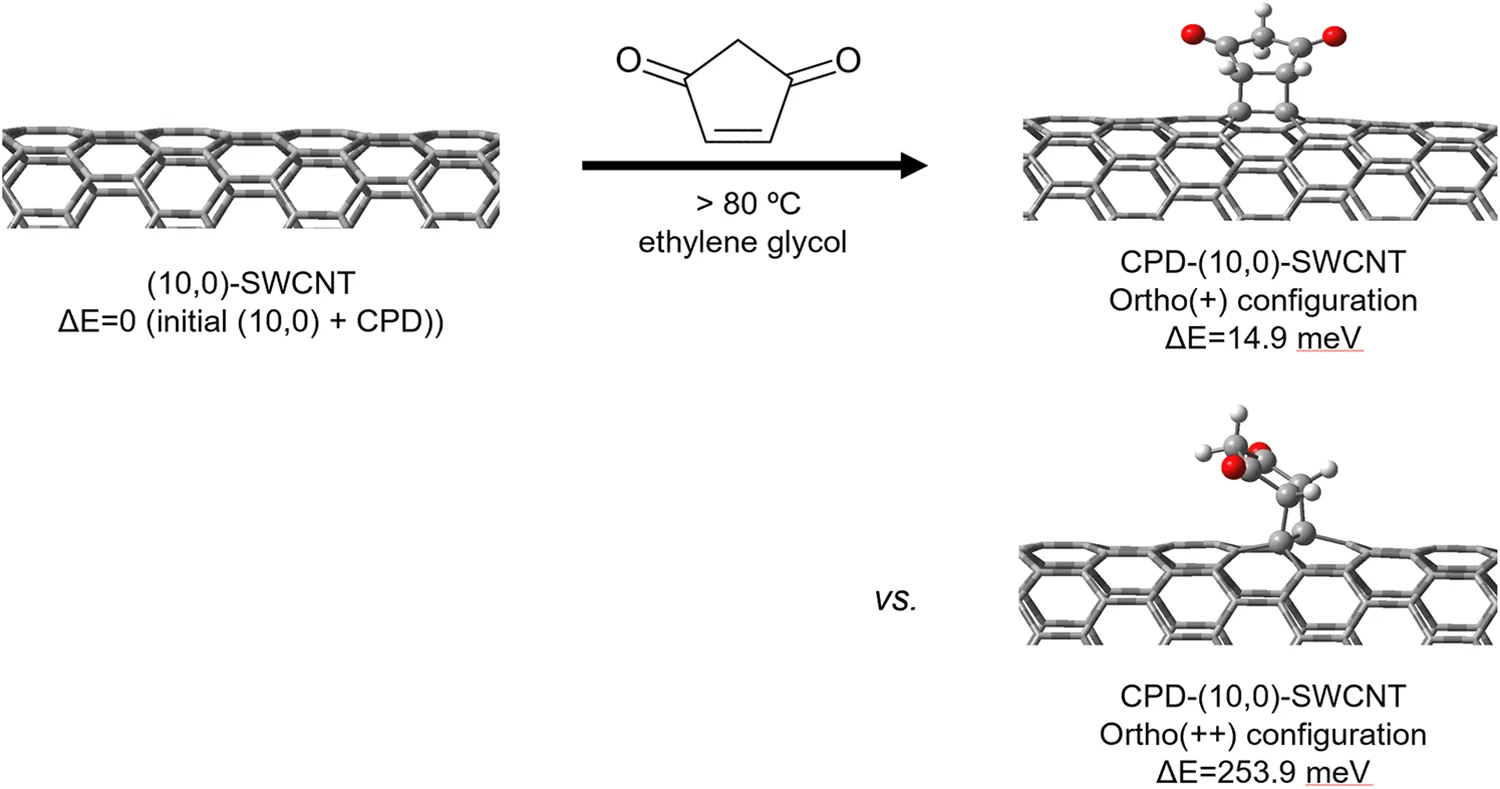
DFT-calculated structures and relative stabilization energies of divalent OCCs in (10,0)-SWCNTs synthetically created through [2 + 2] cycloaddition.

To experimentally validate OCC formation, we functionalized CoMoCat SG65i SWCNTs with CPD using the [2 + 2] cycloaddition reaction in ethylene glycol at elevated temperatures. (10,0) SWCNTs are not abundant in any commercially available carbon nanotube source, and even for the sources in which they are present, including the SWCNT material used here (SG65i, see Supporting Information for details), they are generally a minority species comprising <1% of the total SWCNT mass. This extreme scarcity makes it impossible to directly identify (10,0) OCC emission in ensemble measurements, motivating large-scale reactions, chirality sorting, and single-tube spectroscopy described below.

Absorption spectra and excitation-emission maps of the pristine and functionalized ensembles (Figure S1) show clear spectral changes after CPD reaction, consistent with chemical modification of the nanotubes. For the pristine material, emission arises only from native *E*_11_ transitions of the dominant nanotube chiralities. After CPD functionalization, new emission peaks appear at ≈570 nm excitation with emission in the 1100 nm to 1300 nm spectral window. These redshifted features, assigned as *E*_11_^–^ and *E*_11_^–^*, are consistent with the formation of divalent OCCs in (6,5) nanotubes.^28^ Because (6,5) SWCNTs are a major species in SG65i and we anticipate that all SWCNT species can react under similar conditions, the (6,5) OCC emission serves as a reliable indicator of successful functionalization within the ensemble, even though the minor (10,0) fraction cannot yet be resolved.

To increase the probability of functionalizing and later recovering the rare (10,0) species in the event it required slightly more aggressive conditions than the (6,5), we performed two independent reactions under different conditions (see Supporting Information for details).

These ensemble-level results demonstrate that CPD undergoes [2 + 2] cycloaddition with SWCNTs, producing OCC-related emission. They also highlight the experimental difficulty of probing rare chiralities, such as (10,0), which are present only in trace abundance in typical nanotube sources. Our strategy of large-scale reactions, product pooling, and ATPE sorting provides a general approach for isolating and studying such scarce species. In the following section, we apply this approach to the targeted case of CPD-(10,0) nanotubes, illustrating how symmetry-directed defect engineering can be extended to rare chiralities of interest.

To resolve the spectral signatures of individual chiralities, we employed ATPE^32,33^ to fractionate the CPD-functionalized SG65i material (see Supporting Information for details). This step is essential because (10,0) nanotubes, although targeted, remain at less than 1% abundance in the parent distribution and cannot be identified directly from ensemble measurements. ATPE enabled recovery of purified (6,5), (8,3), and (9,2) fractions, as well as an enriched (8,3) + (10,0) fraction containing ≈40% (10,0) tubes. Together, these fractions allow unambiguous assignment of absorption and PL features to specific chiralities (Figure 2).

**Figure 2:**
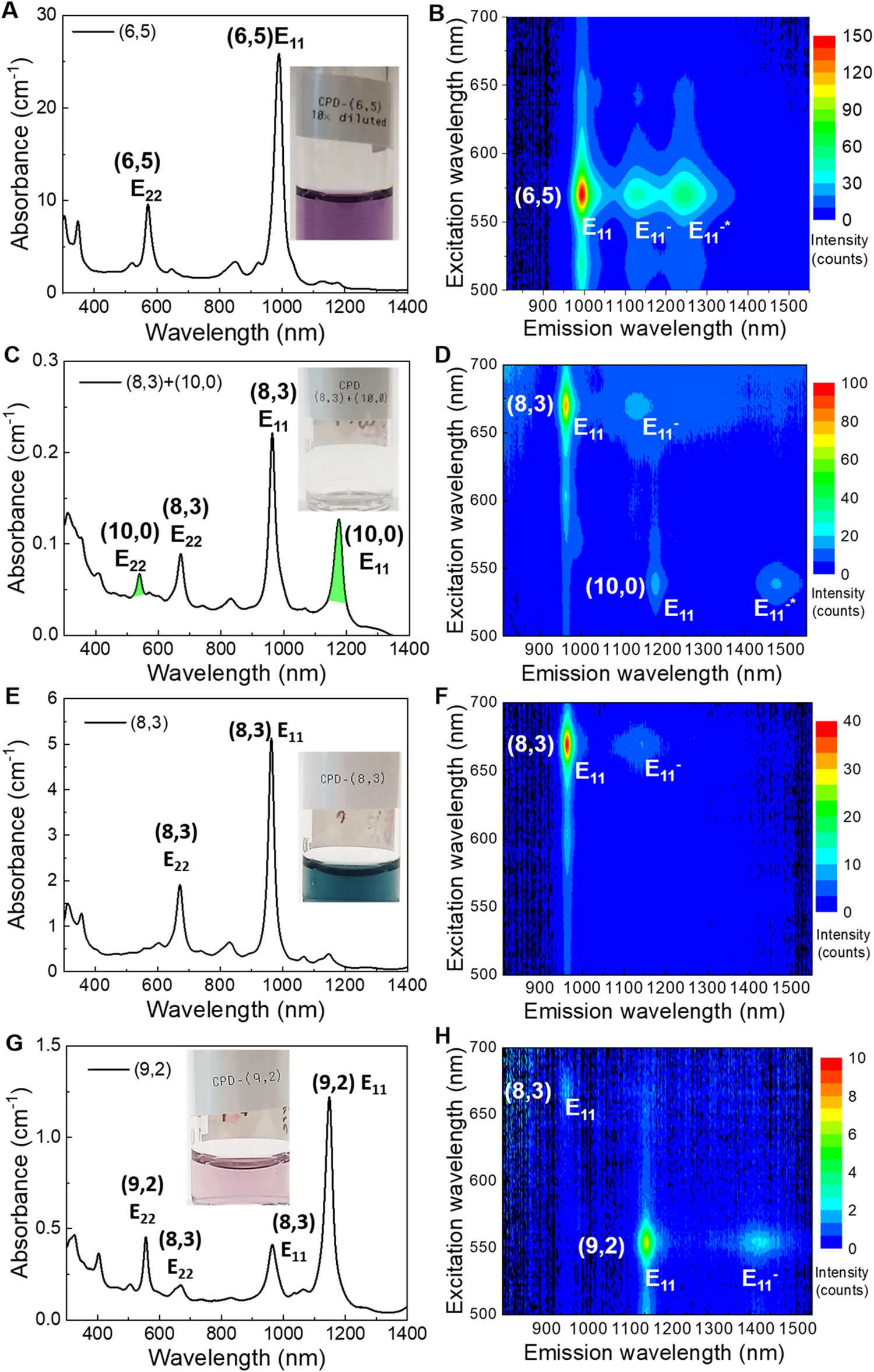
Spectroscopic characterization of chirality-sorted (6,5), (8,3), (9,2), and (10,0) SWCNTs that bear divalent OCCs. (A, B) Absorption and PL map of purified (6,5). (C, D) Absorption and PL map of the (8,3) + (10,0) fraction. (E, F) Absorption and PL map of purified (8,3). (G, H) Absorption and PL map of (9,2) with minor residual (8,3). Insets in (A, C, E, G) show sample photographs.

We obtain a (6,5) fraction with a high chiral purity, as indicated by the absorption spectra (Figure 2A), while still maintaining a strong OCC PL signal (Figure 2B). This result confirms that the presence of the OCC does not have a substantial impact on the results of the ATPE process. While we are unable to obtain a pure (10,0) fraction, we are able to obtain a sample containing only (10,0) and (8,3) as the major species (Figures 2C, 2D), with the (10,0) comprising ≈ 40% of the SWCNT mass, as evaluated by comparing the *E*_11_ absorption peak areas (assuming same absorption cross section for the chiralities). The (10,0) *E*_11_ and *E*_22_ features in the absorption spectrum are highlighted in green in Figure 2C.

The (9,2) fraction provides additional critical control because it is the chirality most likely to be misidentified as (10,0). (9,2) has similar properties to (10,0), including diameter (0.806 nm versus 0.794 nm), *E*_11_ (1138 nm versus 1156 nm in sodium dodecyl sulfate (SDS)/H_2_O), and *E*_22_ (551 nm versus 537 nm, in SDS/H_2_O),^34^ which can make them difficult to distinguish. This challenge is further compounded by the fact that SWCNT PL peaks can shift to tens of nm depending on several factors such as surfactant system or what molecules, if any, are filling the inside of the SWCNT.^35^ Therefore, demonstrating the successful separation of the (9,2) fraction provide a stringent control against spectral misassignment. With negligible (9,2) contamination in the purified sample, we can confidently state that the (10,0) fraction we extracted is indeed (10,0), confirming that the ≈1480 nm OCC emission originates from CPD-(10,0) nanotubes.

The photoluminescence spectra of all four fractions confirm the formation of OCC across all chiralities, with the (8,3) SWCNT exhibiting an OCC peak at 1140 nm and the (10,0) at 1480 nm. Importantly, this assignment is supported by comparison with the purified (8,3) fraction, which shows only the 1140 nm OCC emission, with no long-wavelength feature near 1480 nm.

Although ATPE enriches (10,0) nanotubes, the recovered fraction still contains substantial contributions from other chiralities such as (8,3). To unambiguously identify OCC emission from (10,0), we employ single-tube PL imaging combined with multi-step spectral screening, conducted on spin-coated SWCNTs on a silicon–gold-polystyrene substrate. The spectra from CPD-(10,0) nanotubes were required to (i) exhibit a single *E*_11_ peak within 1180–1200 nm, (ii) show no additional peaks between 1050–1300 nm indicative of other chiralities, and (iii) contain a defect-related emission peak between 1300–1600 nm exceeding a defined intensity threshold. From initially detected emitters, 24 (10,0) nanotubes satisfied all screening conditions and were used for detailed characterization (see Supporting Information for details).

To isolate clean CPD-(10,0) emission, the influence of OCC peaks or *E*_11_ peaks from other chiral species is eliminated by implementing a multiple-step screening process, from which clean (10,0) tube emission spectra are extracted (Figure 3). Firstly, we identify spectra with bright emission features by integrating the full spectrum at all points. Figures 3A–D show examples of the selected spectra Figure 3A shows a single emission peak outside (10,0) *E*_11_ spectral window, while Figure 3B represents a spectrum containing emission from multiple tubes. In comparison, Figures 3C and 3D exhibit single peaks in the (10,0) *E*_11_ spectral window.

**Figure 3:**
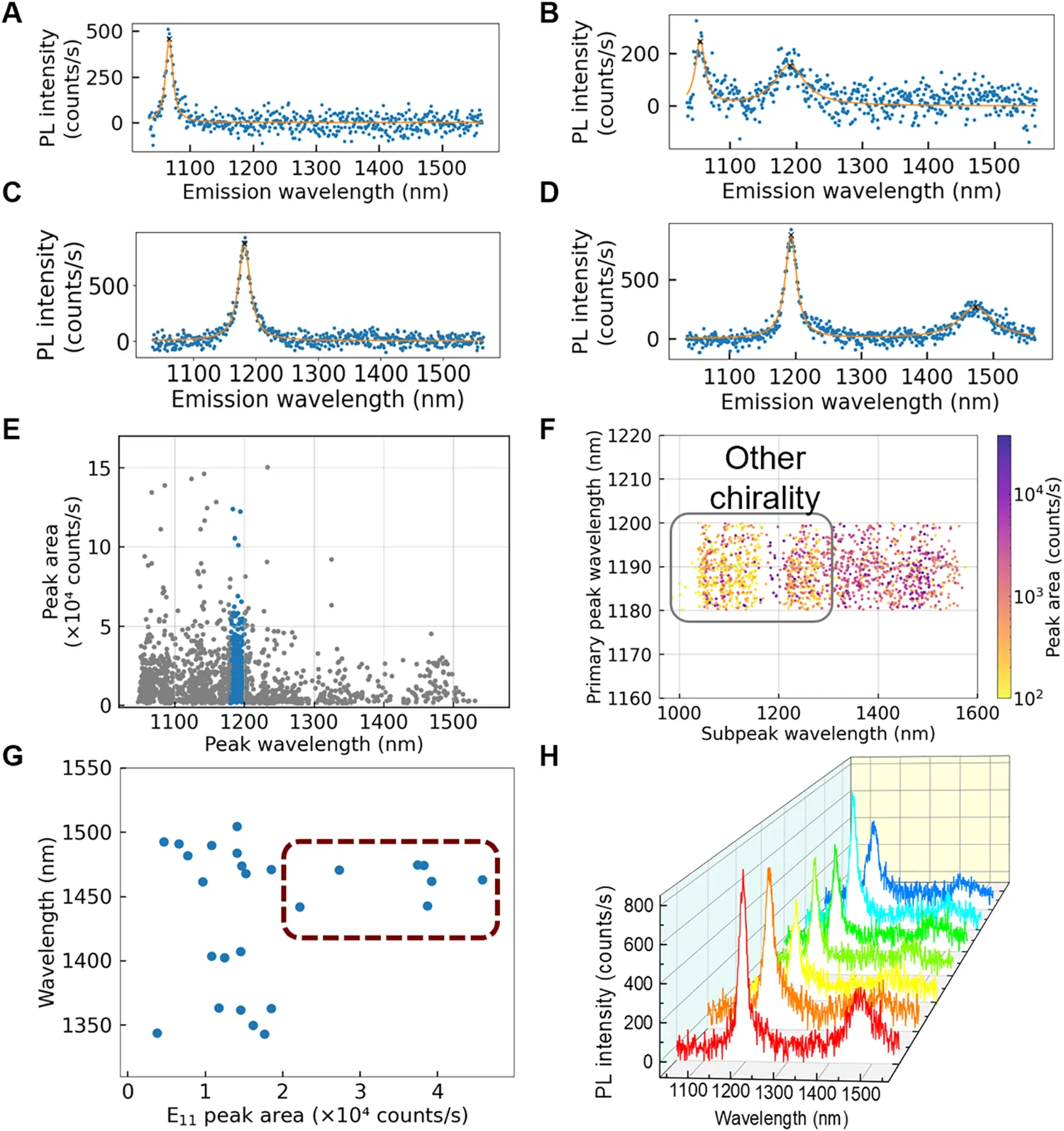
Multi-step spectral screening demonstrates uniform OCC emission from CPD-(10,0) SWCNTs. PL spectra are extracted from 2D PL maps of spin-coated CPD-(10,0) nanotubes. (A–D) Representative PL spectra illustrating different screening outcomes: (A) spectra without the (10,0) *E*_11_ peak, (B) spectra showing emission peaks from (10,0) and other chiral nanotubes, (C) spectra exhibiting only the (10,0) *E*_11_ peak without OCC emission, and (D) spectra showing both OCC and (10,0) *E*_11_ peaks, representing the final set of selected individual CPD-(10,0) nanotubes. (E) The scatter plot distribution of emission peak parameters in the 1050 nm to 1550 nm range. Spectra are filtered to select those with a (10,0) *E*_11_ emission peak in the 1180 nm to 1200 nm range, which are shown as blue scatter points. (F) Scatter plot of subpeak wavelength versus primary peak wavelength after excluding the (10,0) *E*_11_ peak. Spectra with peaks below 1300 nm are filtered out, as highlighted in the gray box. (G) Scatter plot showing the OCC peak emission wavelength versus the *E*_11_ peak area. The plots with *E*_11_ emission peak areas greater than 20,000 counts/s are highlighted in the red box. (H) Corresponding PL spectra from bright *E*_11_ emission regions, where the *E*_11_ peak area is larger than 20,000 counts/s.

Each spectrum is fitted with a multi-Lorentzian function and the resulting peak area is plotted as a scatter plot in Figure 3E. As the second step in the screening process, we select 380 spectra containing the *E*_11_ emission from (10,0) tubes shown as blue dots in Figure 3E. This step filters out spectra consisting only of emission from other chiralities, such as that shown in Figure 3A. While this criterion ensures the selection of spectra containing clear (10,0) *E*_11_ emission, it may also inadvertently exclude cases where defect-related emission dominates and the *E*_11_ peak is weak, which is often observed in shorter nanotubes. Consequently, the apparent fraction of functionalized (10,0) tubes could be underestimated compared with the absorbance-based abundance.

In the third screening step, we exclude spectra containing emission peaks from other chirality tubes by inspecting the subpeaks, which we define as all peaks other than the main (10,0) *E*_11_ peak. We plot the wavelengths and peak areas of all the subpeaks as a function of the main peak wavelength in Figure 3F. In the scatter plot, the subpeaks in the 1050 nm to 1300 nm range can be mostly attributed to emission from nanotubes of other chirality. These spectra are excluded, resulting in approximately 130 spectra. Since the selected spectra only contain PL signals from individual (10,0) tubes, we can undoubtedly attribute their subpeaks in the 1300 nm to 1600 nm region to OCC emission of the CPD-(10,0) tubes.

Our experiments are designed to produce a low density of OCCs, ideally one per nanotube, which necessarily results in a stochastic distribution with many tubes remaining unfunctionalized. Consequently, many (10,0) spectra lack detectable OCC peaks, as shown in Figure 3C. In the final screening step, we identify spectra exhibiting OCC emission in the 1300 nm to 1600 nm range. From the 24 spectra that satisfied all the screening criteria, we extracted the OCC peak properties (Figure S4). Although OCCs are generally expected to exhibit higher intrinsic quantum yields than pristine *E*_11_ excitons, the experimentally observed defect emission intensity also depends on exciton diffusion and trapping efficiency at room temperature. Therefore, a relatively weak *E*_11_^–^* intensity does not necessarily indicate a lower intrinsic radiative efficiency of the OCC state, particularly in the case of sparsely functionalized nanotubes containing only one or a few defect sites. Based on the emission wavelength, we classify the OCC peaks into two types: *E*_11_^–^ peaks (1350 nm to 1400 nm) and *E*_11_^–^* peaks (1450 nm to 1500 nm). Among the 24 individual functionalized (10,0) nanotubes that satisfied all screening criteria, 22 exhibited only a single defect-related emission peak, whereas only 2 nanotubes showed two distinguishable defect emission peaks (Figure S4). This observation indicates a low OCC density in the analyzed nanotube population and suggests that the measured spectra are generally dominated by a single emissive OCC within the optical resolution of our measurements. According to the OCC peak emission from previous measured PLE images of the (10,0) + (8,3) fraction (Figure 2D), we expect the *E*_11_^–^* peak to be dominant for CPD-(10,0) tubes. Consistent with our DFT calculations (Figure 1), which predict that the ortho­(+) configuration is the most stable and has the lower emission energy, we assign it to the observed *E*_11_^–^* peak to emission from this OCC configuration.

There are, however, a considerable number of spectra with an *E*_11_^–^ peak rather than *E*_11_^–^*. Given the appearance of the *E*_11_^–^ peak, we examine potential factors influencing OCC emission characteristics by plotting the relationship between the *E*_11_ emission area and the OCC peak emission wavelength (Figure 3G). For spectra with a small *E*_11_ peak area (<20,000 counts/s), OCC emission peaks appear with a broad distribution, including both *E*_11_^–^ and *E*_11_^–^* emission. In contrast, spectra with a large *E*_11_ peak area (≥20000 counts/s) exhibit OCC emission confined to a more limited range of 1450 nm to 1500 nm, corresponding to the *E*_11_^–^* emission. The PL spectra of the tubes with brighter *E*_11_ signals are shown in Figure 3H.

The correlation between *E*_11_ emission intensity and OCC peak distribution suggests that the nanotube quality has an influence. Previous studies have shown that the PL brightness of SWCNTs correlates with tube length and defect density, with defects typically acting as non-radiative quenching sites.^36,37^ As described in the Methods section, sonication is applied during both purification and washing to obtain a well-dispersed solution of CPD-(10,0) nanotubes. However, the sonication process inevitably introduces sidewall damage or even tube breakage.^28^ In addition, the experimentally observed defect distribution is likely influenced not only by the thermodynamic preference predicted by DFT calculations but also byreaction kinetics and local environment during functionalization, including variations in the initial CNT structure and the local chemical environment. These factors may give rise to a small fraction of additional OCC configurations under experimental conditions. Nevertheless, the apparent difference between the relative *E*_11_^–^/*E*_11_^–^* populations obtained from ensemble and single-nanotube measurements should be interpreted with caution because the two techniques probe fundamentally different statistical quantities. Ensemble PL represents an intensity-weighted average over the entire nanotube population, whereas single-nanotube spectroscopy reflects the occurrence of individual emissive nanotubes. As shown in Figure 3H, *E*_11_^–^* emission peak area exceeds those of *E*_11_^–^, indicating that *E*_11_^–^* remains the predominant defect emission. In addition, inspection of the individual functionalized nanotube spectra (Figure S4) reveals that nanotubes exhibiting *E*_11_^–^ emission are, in most cases, accompanied by an *E*_11_^–^* peak with comparable or even higher intensity. Consequently, these nanotubes contribute to the occurrence statistics of *E*_11_^–^ in the single-nanotube analysis while contributing predominantly to the *E*_11_^–^* intensity in the ensemble measurement. Taken together, we infer that the uniform OCC peak wavelength observed in the brighter tubes is associated with better crystallinity and longer lengths of the functionalized nanotubes. It implies that monodispersed quantum emitters would be obtained if thorough sonication could be circumvented.

Our spectroscopic analysis supports the predominance of a specific OCC configuration in CPD-functionalized (10,0) nanotubes. Direct structural characterization of individual OCCs represents an important future direction. Such approaches would provide complementary information on the atomic-scale defect structures and further clarify the relationship between OCC configurations and their optical emission properties.

Next, we perform time-resolved and photon correlation measurements to characterize *E*_11_^–^* emission from the CPD-(10,0) tubes. A tube exhibiting both a strong *E*_11_ emission signal (peak area ≥ 20,000 counts/s) and *E*_11_^–^* emission peak is selected from those shown in Figure 3H. The PL spectrum of this functionalized tube measured with an extended integration time is shown in Figure 4A. Time-resolved PL measurements are then performed using pulsed laser excitation. In Figure 4B, the red curve is obtained without a long-pass filter; therefore, the signal is dominated by *E*_11_ emission. A lifetime of 37 ps is obtained by fitting to a single exponential convolved with the instrument response function, which is consistent with ensemble measurements.^38^ The gray curve shows data taken with a long-pass filter having a cut-on wavelength of 1450 nm, collecting only the *E*_11_^–^* emission. We perform a reconvolution fit using a biexponential decay function

$$I\left(t\right)=A_{1}\cdot \exp\left(-t/\tau_{1}\right)+A_{2}\cdot \exp\left(-t/\tau_{2}\right)$$

where *I*(*t*) is the PL intensity, *t* is the delay time after excitation, τ represents the decay time, and *A* is the amplitude for each component, with subscripts 1 and 2 denoting the short- and long-lifetime components, respectively. Compared to the typical measurement of solution-dispersed tubes,^38^ the extracted *E*_11_^–^* emission lifetimes τ_1_ = 63 ps and τ_2_ = 402 ps are consistent with the presence of an OCC in the CPD-(10,0) SWCNT.

**Figure 4:**
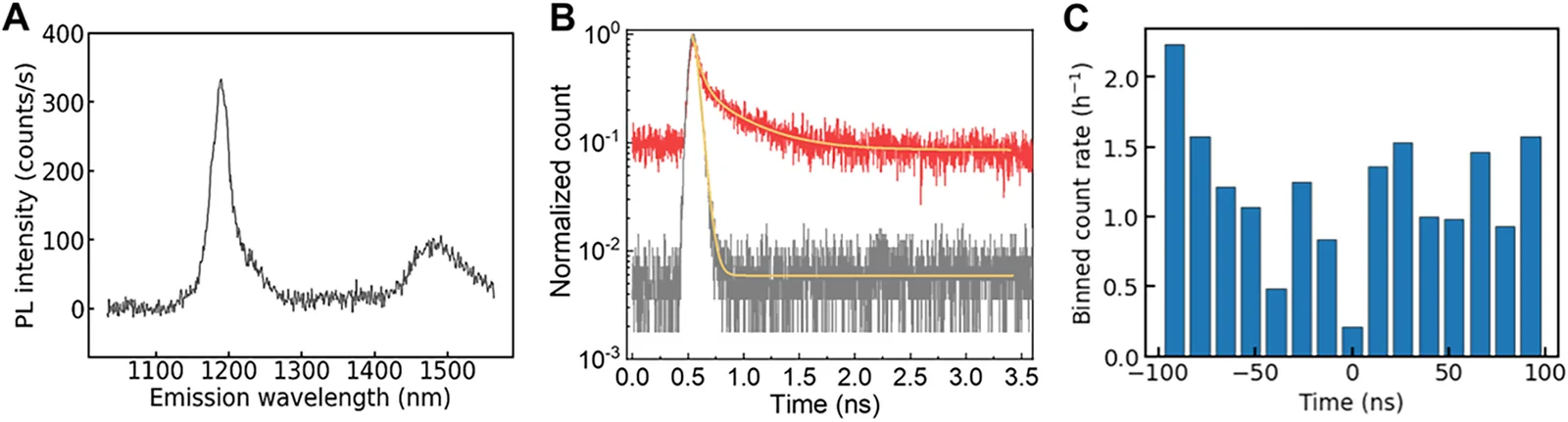
Time-resolved and photon correlation measurements of CPD-(10,0) SWCNTs. (A) PL spectrum of the CPD-(10,0) used for single photon measurement showing the *E*_11_ peak at ∼1190 nm and *E*_11_^–^* peak at ∼1450 nm. The excitation was performed at 880 nm, 30 μW, and an integration time of 2000 ms. The polarization angle of the excitation laser is 60°, which is parallel to the tube axis. (B) Lifetime measurement of the *E*_11_^–^* emission peak (red) and *E*_11_ emission peak (gray), measured under 880 nm excitation at 250 nW and a polarization angle of 100°, with a total acquisition time of 300 s. The lifetime of *E*_11_^–^* emission peak is separately obtained using a 1450 nm long pass filter. (C) *g* ^(2)^(0) measurement showing the emitting defect is a single photon emitter, recorded under 880 nm excitation at 250 nW, polarization angle 60°, and a total integration time of 8 h.

Photon correlation measurements are also performed for *E*_11_^–^* emission on the same CPD-(10,0) tube (see details in the Methods section). As shown in Figure 4C, the second-order correlation function at zero delay *g* ^(2)^(0) is measured to be 0.17, confirming single-photon emission at room temperature.

## Conclusion

In summary, we demonstrated that symmetry-directed [2 + 2] cycloaddition provides a synthetic route to significantly reduce the configurational heterogeneity of organic color centers (OCCs) in carbon nanotubes. Density functional theory predicted that the high mirror symmetry of the (10,0) zigzag lattice reduces the number of unique divalent defect geometries to two, with an energy gap of >200 meV, favoring the selective formation of a single defect configuration. Guided by this prediction, we functionalized SWCNT ensembles with 4-cyclopentene-1,3-dione, enriched the (10,0) species, and identified its OCC emission at approximately 1480 nm (a shift of 220 meV from *E*_11_). These quantum emitters exhibit a substantially narrowed distribution of defect-state emission, consistent with the predominance of a single OCC geometry. The correlation between the nanotube *E*_11_ PL brightness and OCC peak distribution suggests that higher-quality tubes consistently exhibit brighter OCC emission. Subsequent lifetime and photon correlation measurements confirm room-temperature single-photon emission from these OCCs. The results highlight the importance of preserving nanotube crystallinity during processing to further improve emitter uniformity, while demonstrating the potential of divalent OCCs on high-symmetry SWCNTs as promising candidates for atomically defined quantum light sources in scalable quantum photonics.

## Methods

Certain equipment, instruments, software, or materials, commercial or non-commercial, are identified in this paper in order to specify the experimental procedure adequately. Such identification is not intended to imply recommendation or endorsement of any product or service by the National Institute of Standards and Technology (NIST), nor is it intended to imply that the materials or equipment identified are necessarily the best available for the purpose.

### Creation of OCCs in SWCNT

We prepare OCC functionalized carbon nanotube by dispersing CoMoCat SG65i SWCNT powder (Lot# L64, CHASM), which we previously confirmed to contain some fraction of (10,0) SWCNT, into ethylene glycol (Fisher Scientific) at a concentration of 0.2 mg/mL by tip sonication for 30 min at a power of 1 W/mL (Misonix S-4000, 417-A tip). 25 mL of this solution was added to a 25 mL round-bottom flask, along with 5 mg (50 mg) of 4-cyclopentene-1,3-dione (CPD, 95%, Aldrich), for a mass ratio of 1:1 (10:1) CPD:SWCNT. The mixture was heated in an oil bath at 80 °C (105 °C) for 24 h. After the reaction, the SWCNTs were collected by vacuum filtration through a poly­(tetrafluoroethylene) (PTFE) membrane (Millipore JGWP, 0.2 μm pore) and washed with 15 mL of ethanol and 15 mL of water to remove excess ethylene glycol and unreacted CPD. To confirm the reaction was successful, a small amount (≈0.1 mg) was redispersed into 10 g/L sodium deoxycholate (DOC, BioXtra ≥98.0%, Sigma-Aldrich) in D_2_O (99.8%, Cambridge Isotope Labs) by tip sonication, and the photoluminescence spectrum was collected.

### Chirality Sorting by Aqueous Two-Phase Extraction

The CPD-functionalized SG65i powder was redispersed in a 20 g/L DOC/H_2_O solution to a concentration of 1 mg/mL using tip sonication at 1 W/mL for 40 min. The resulting dispersion was purified, concentrated, and separated into chirality-enriched fractions using the aqueous two-phase extraction (ATPE) method, following the methods previously described in the literature.^32,33^ Similar fractions enriched in (10,0) were combined from different separation experiments and subjected to the addition of (*n*,*m*) selective ATPE to resolve the final (10,0) sample. Repeated ultrafiltration was used to remove the ATPE polymers, concentrate the SWCNTs, and set the DOC concentration to 10 g/L.

### Computational Methods

Density functional theory (DFT) calculations were performed using a 6 nm (10,0) SWCNT. The nanotube ends were capped with hydrogen atoms and three methylene groups on each end to suppress spurious edge-localized states.^39^ Geometry optimization and time-dependent DFT calculations were performed at the B3LYP/STO-3G level of theory using the Gaussian 16 software package.^40^ Four model systems were evaluated: pristine (10,0), the CPD molecule by itself, and CPD-functionalized (10,0) in two possible configurations (ortho­(+) and ortho­(++)). The relative stabilization energies for the ortho­(+) and ortho­(++) configurations were obtained by comparing the total electronic energies of the functionalized nanotube models to the sum of the energies of the pristine (10,0) and the CPD molecule. The energy shifts in defect emission were determined within the same functionalized model rather than against the *E*_11_ of a pristine nanotube, under the assumption that OCC-induced quantum confinement affects both *E*_11_ and the defect emission energy to a similar degree. We note that TD-DFT systematically overestimates the energy gap between *E*_11_ and the OCC emission. However, this error is expected to result in a similar shift for all OCC configurations, preserving the relative energetic ordering.^41^

### Substrate Preparation

(8,3) + (10,0) SWCNT in 10 g/L DOC/D_2_O were spin-coated onto a silicon–gold-polystyrene substrate at 315 rad/s (3 kRPM), then washed with isopropyl alcohol to remove the excess DOC surfactant. To protect the sample from humidity and oxygen in air, the SWCNTs were encased in polystyrene (MW ≈ 280 kDa, Aldrich) by spin-coating a second layer on top of the SWCNTs from a 50 g/L polystyrene in eucalyptol (99%, Sigma-Aldrich) solution, followed by heating in a vacuum oven overnight at 100 °C to remove excess eucalyptol.

### PL Measurements

PL measurements are performed on the deposited CPD-SWCNTs in a home-built confocal microscopy system. A wavelength-tunable Ti:sapphire laser with continuous-wave output is used. The excitation laser beam with a power of 10 μW to 100 μW and linear polarization parallel to the SWCNT axis is focused onto the sample by an objective lens with a numerical aperture of 0.80 and a focal length of 1.80 mm. The reflected beam and PL from the sample are collected by the same objective lens and separated by a dichroic mirror. A Si photodiode detects the reflected beam to visualize the trenches on the substrate, and PL is collected by an InGaAs photodiode array attached to a spectrometer. Unless otherwise noted, all measurements are carried out at room temperature.

A 400 μm × 400 μm area is scanned with 1 μm steps by an 850 nm excitation laser, and PL signals from deposited SWCNTs are collected (Figure S2A). Although the direct optical coupling of 850 nm excitation to the *E*_11_ transition of (10,0) nanotubes is relatively weak, it lies close to the *E*_11_ 2u excited state and thus provides sufficient excitation efficiency (Figure S2B). However, this wavelength can also be resonant with the *E*_22_ transitions of other chiral species, leading to PL signals from multiple (*n*,*m*) nanotubes. Therefore, we need to spectrally screen the emission.

### Lifetime and Photon Correlation Measurements

A laser output of approximately 100 fs pulses and a repetition rate of 76 MHz is used. The excitation laser beam is focused onto the sample by an objective lens with a numerical aperture of 0.85 and a focal length of 1.80 mm. The data are collected by a fiber-coupled superconducting single-photon detector (EOS210; Single Quantum) and a time-correlated single-photon counting module (QuTAG, qutools). Photon correlation measurements are carried out using a Hanbury–Brown–Twiss setup equipped with a 50:50 fiber coupler. The binned count-rate histogram is constructed by first subtracting the dark counts averaged from regions outside each coincidence peak. The counts within each coincidence peak are then integrated, and the resulting values are divided by the experimental exposure time to obtain the count rate for the statistical analysis of photon correlation.

## Supplementary Material



## Notes

Spectral characterization of pristine and CPD-functionalized SG65i SECNTs; photoluminescence maps of CPD-functionalized SG65i SWCNTs used for aqueous two-phase extraction separation; single-tube photoluminescence imaging and spectra characterization of CPD-(10,0) SWCNTs; additional photoluminescence spectra of identified CPD-(10,0) SWCNTs exhibiting OCC emissions (PDF)
