# Supplementary material for: Symmetry-Directed Engineering of Quantum Defects: Toward Monodispersed Organic Color Centers in Carbon Nanotubes
Source: ACS Nano. 2026 Oct 15;20(42):28217–25. doi: 10.1021/acsnano.6c07965 (PMC13718604; doi:10.1021/acsnano.6c07965)
Supplement: Supplementary file 1 [file nn6c07965_si_001.pdf]

Supporting Information

Symmetry-Directed Engineering of Quantum Defects: Toward Monodispersed Organic Color Centers in Carbon Nanotubes

Mengyue Wang[1,2♯], Jacob Fortner[3,4♯], Benjamin Barnes[5], Daichi Kozawa[1,2,6], Chee Fai Fong[1,2], Jeffrey A. Fagan[5], Yuichiro K. Kato[1,2*], and YuHuang Wang[3,4,7*]

1. Quantum Optoelectronics Research Team, RIKEN Center for Advanced Photonics, Wako, Saitama, 351-0198, Japan

2. Nanoscale Quantum Photonics Laboratory, RIKEN Pioneering Research Institute, Wako, Saitama 351-0198, Japan

3. Chemical Physics Program, Institute for Physical Science and Technology, University of Maryland, College Park, Maryland 20742, United States

4. Department of Chemistry and Biochemistry, University of Maryland, College Park, Maryland 20742, United States

5. Materials Science and Engineering Division, National Institute of Standards and Technology, Gaithersburg, Maryland 20899, United States

6. Research Center for Materials Nanoarchitectonics, National Institute for Materials Science, Tsukuba, Ibaraki 305-0044, Japan

7. Maryland NanoCenter, University of Maryland, College Park, Maryland 20742, United States

♯ These two authors contributed equally to this work.

Correspondence should be addressed to Y.K.K. (yuichiro.kato@riken.jp) or Y.H.W. (yhw@umd.edu)

Acronyms:

CPD: 4-cyclopentene-1,3-dione

CNT: carbon nanotube

SWCNT: single-wall carbon nanotube

DOC: sodium deoxycholate

PL: photoluminescence

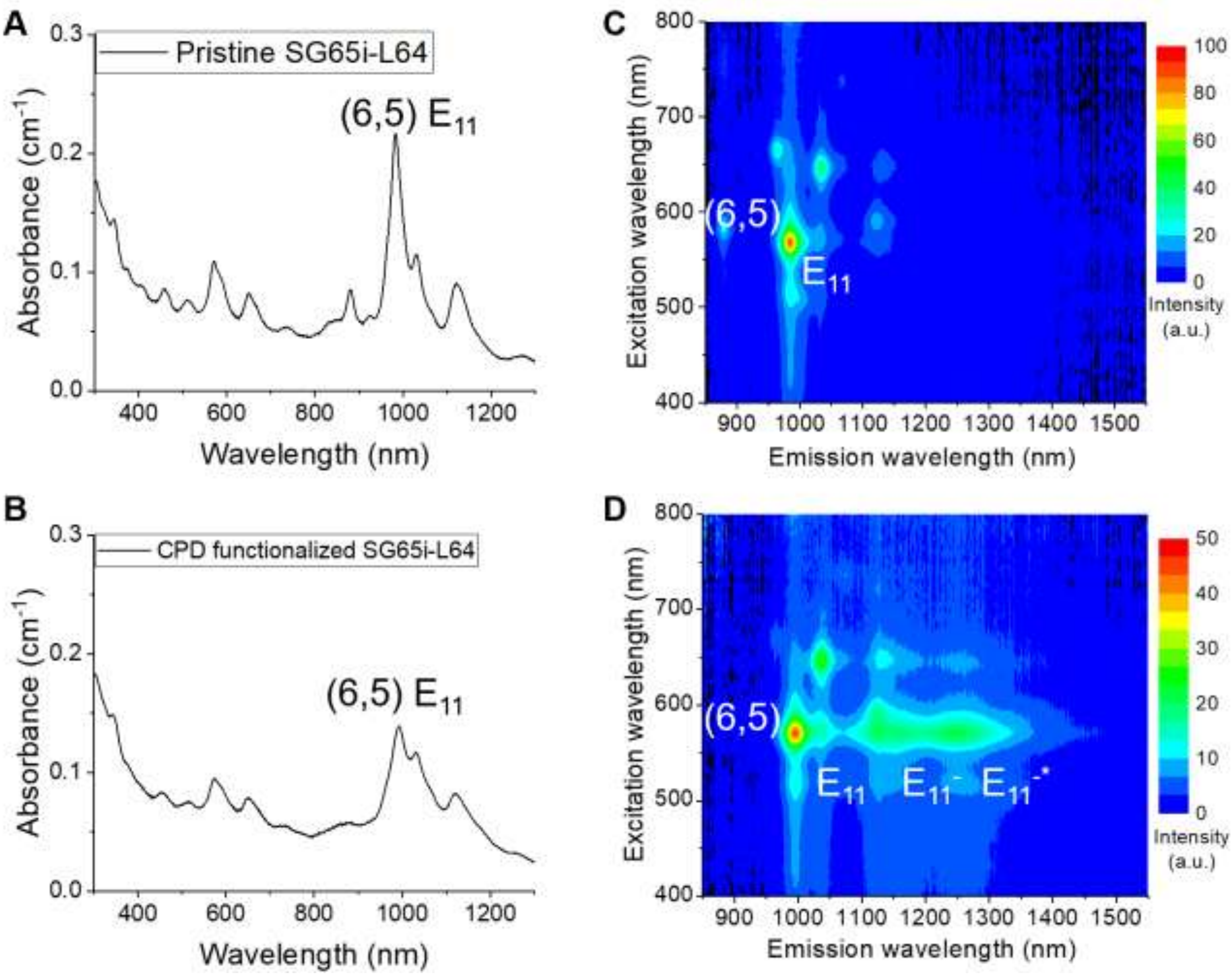

Figure S1. Spectral characterization of synthesized OCCs-containing SWCNTs. (A) The absorption spectrum of pristine SG65i SWCNTs. (B) The absorption spectrum of 4-cylopentene-1,3-dione-functionalized SWCNTs. (C) Excitation-emission map of pristine SG65i SWCNTs. (D) Excitation-emission map of 4-cylopentene-1,3-dione-functionalized SG65i SWCNTs. The SWCNTs are individually dispersed in 10 g/L DOC/D_2O.

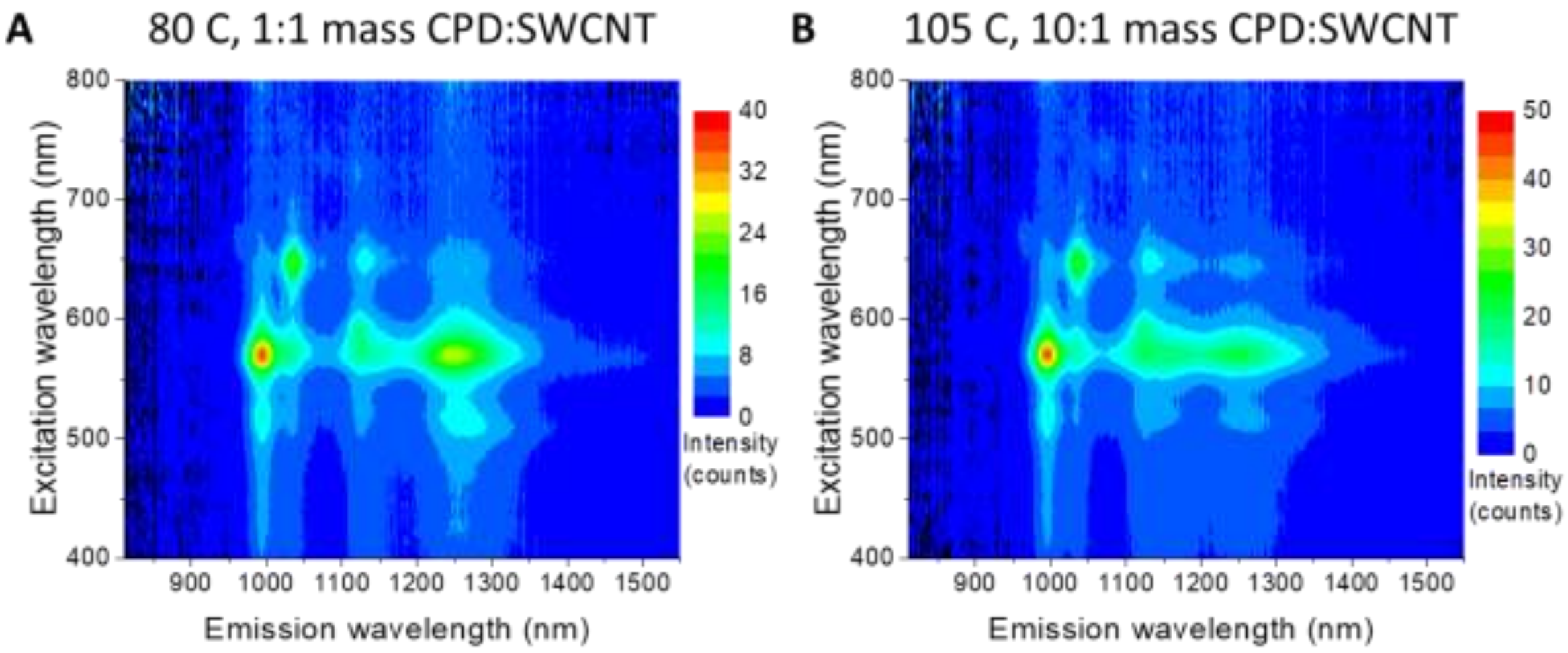

Figure S2. Photoluminescence (PL) maps of CPD-functionalized SG65i SWCNTs used as starting material for aqueous two-phase extraction (ATPE) separation. (A) Sample prepared with a 1:1 CPD-to-CNT mass ratio, heated at 80 °C for 24 h. (B) Sample prepared with a 10:1 CPD-to-CNT mass ratio, heated at 105 °C for 24 h. After filtering the CPD-SWCNT from the ethylene glycol, a small amount (≈ 0.1 mg) of the resulting solid was redispersed in 10 g/L DOC/D_2O by tip sonication. Because (10,0) chirality constitutes < 1 % of the starting SG65i material, the two 5 mg samples were combined to provide sufficient material for subsequent ATPE separation of the (10,0) SWCNTs.

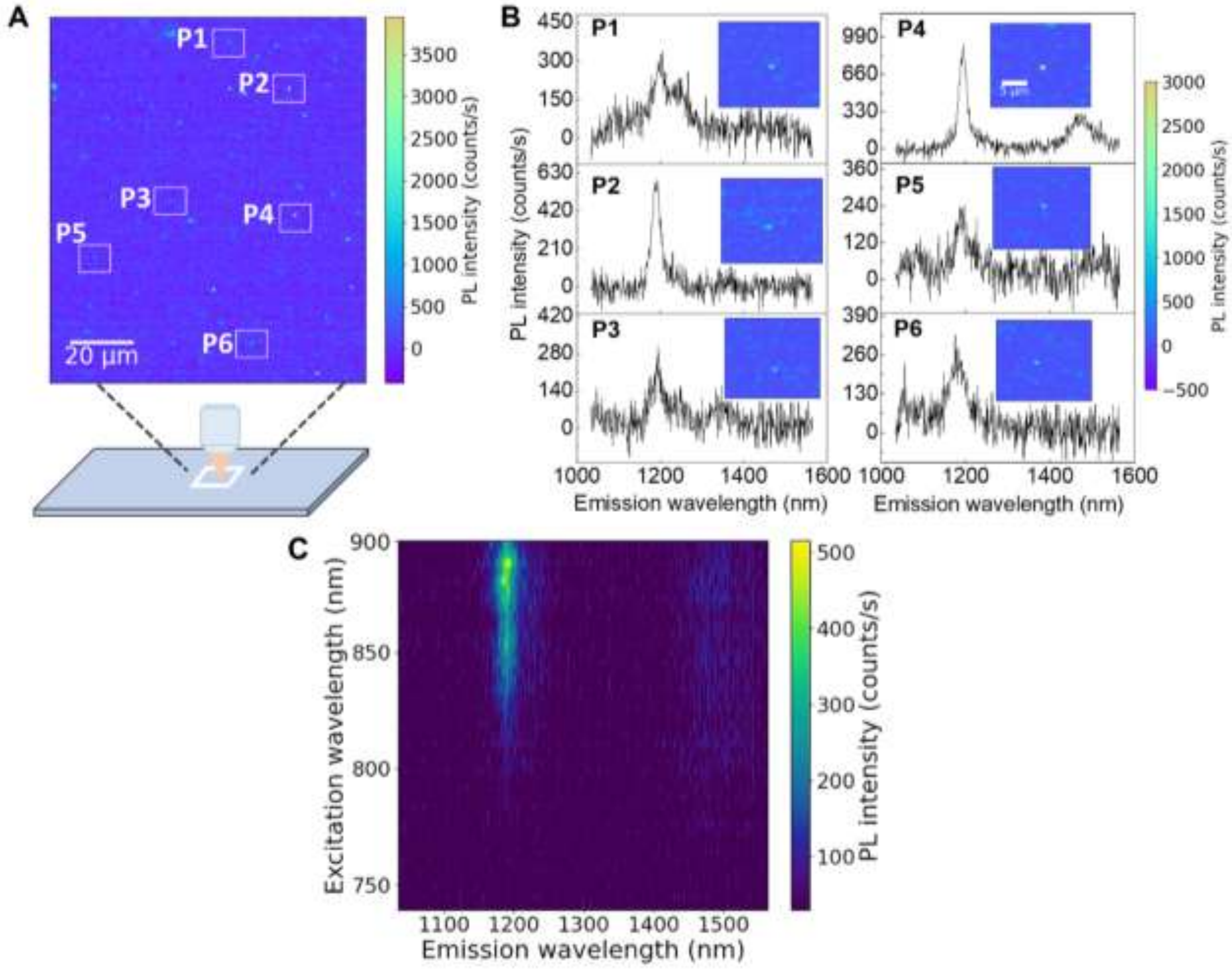

Figure S3. Single-tube PL imaging of CPD-(10,0) SWCNTs with measured positions (P(i)) labelled. (A) PL image of CPD-(10,0) spin-coated on a Si chip, acquired under 850 nm excitation at 100 µW with a polarization angle of 90° and an integration time of 200 ms. The scale bar represents 20 µm. (B) Representative PL spectra from the bright spots in (A). The insets show the corresponding 2D PL images, indicating that each spectrum originates from the highlighted bright region. The scale bar represents 5 µm. (C) Photoluminescence excitation (PLE) map measured from an individual CPD-(10,0) tube under an excitation power of 30 µW. The excitation wavelength was tuned from 740 nm to 900 nm.

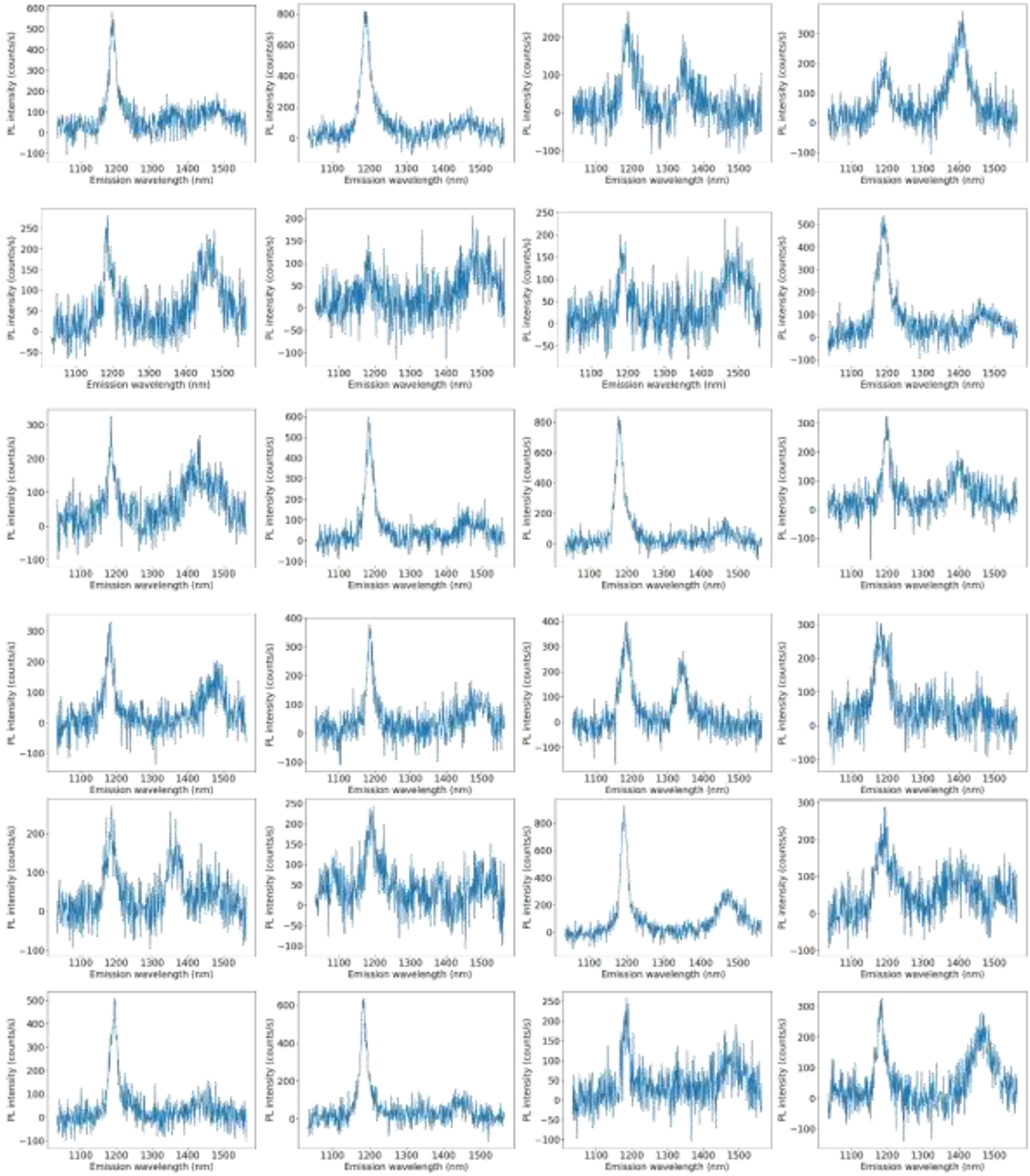

Figure S4. All identified CPD-(10,0) spectra exhibiting OCC emissions in the 1300 nm to 1600 nm range with a peak area greater than 900 counts/s.
